# The ubiquitin-proteasome system is required for African swine fever replication

**DOI:** 10.1371/journal.pone.0189741

**Published:** 2017-12-15

**Authors:** Lucía Barrado-Gil, Inmaculada Galindo, Diego Martínez-Alonso, Sergio Viedma, Covadonga Alonso

**Affiliations:** Department of Biotechnology, Instituto Nacional de Investigación y Tecnología Agraria y Alimentaria, INIA, Madrid, Spain; Meharry Medical College, UNITED STATES

## Abstract

Several viruses manipulate the ubiquitin-proteasome system (UPS) to initiate a productive infection. Determined viral proteins are able to change the host’s ubiquitin machinery and some viruses even encode their own ubiquitinating or deubiquitinating enzymes. African swine fever virus (ASFV) encodes a gene homologous to the E2 ubiquitin conjugating (UBC) enzyme. The viral ubiquitin-conjugating enzyme (UBCv1) is expressed throughout ASFV infection and accumulates at late times post infection. UBCv is also present in the viral particle suggesting that the ubiquitin-proteasome pathway could play an important role at early ASFV infection. We determined that inhibition of the final stage of the ubiquitin-proteasome pathway blocked a post-internalization step in ASFV replication in Vero cells. Under proteasome inhibition, ASF viral genome replication, late gene expression and viral production were severely reduced. Also, ASFV enhanced proteasome activity at late times and the accumulation of polyubiquitinated proteins surrounding viral factories. Core-associated and/or viral proteins involved in DNA replication may be targets for the ubiquitin-proteasome pathway that could possibly assist virus uncoating at final core breakdown and viral DNA release. At later steps, polyubiquitinated proteins at viral factories could exert regulatory roles in cell signaling.

## Introduction

The ubiquitin-proteasome (UP) system is an important non-lysosomal protein degradation system in eukaryotic cells [1]. Ubiquitin is a small (76 amino acids; aa) and highly conserved protein present in almost all eukaryotic cells. The addition of these few aa, so-called ubiquitination (UB), is the signal to direct proteins to the proteasome [2]. Ubiquitination of particular targets is involved in a diversity of cellular processes such as transcription, transduction, regulation of the immune response, control of cell division [3], development, endocytosis [4], cellular trafficking [5], and cell survival control [6]. The length (poly- versus monoubiquitination) and the site of ubiquitination define whether the protein would be sent to the proteasome, or if ubiquitination regulates any target protein activity. The first characterised signal for proteasome-mediated degradation was the polyubiquitin chains linked to Lysine in position 48 [7]. The binding of monoubiquitin or non-Lys 48 chain linkages onto substrates has several consequences [8]. On the other hand, Lys 63-linked polyubiquitin chains, participate in the oxidative response and the regulation of innate immunity signalling pathways [9–11]. The conjugation of ubiquitin to substrates involves three steps. First, the ubiquitin-activating enzyme (E1) stablishes a thiol ester bond with the C-terminal Gly of ubiquitin, becoming active for nucleophilic attack. The activated ubiquitin is then transferred to an ubiquitin conjugating enzyme (E2) by transesterification. Finally it is attached, usually with an isopeptide bond, to the substrate protein by the action of a ubiquitin ligase (E3), critical for the specificity and the efficiency of the reaction. Furthermore, nearly 100 de-ubiquitylating enzymes (DUB) may participate in the regulation of this pathway [12,13].

This complex system can be manipulated by viruses to pursue their life cycles relying on such specific interactions with regulatory systems of the target cells. Viruses have developed effective strategies to control the UPS for virus replication and some viruses even encode viral proteins that are able to ubiquitinate or deubiquitinate proteins [14,15].

African Swine Fever Virus (ASFV) is a double-stranded DNA virus with icosahedral morphology that belongs to the *Asfarviridae* family [16]. ASFV particles are organized in diverse concentric domains. The inner core is the nucleoid, covered with the core shell, then an inner lipid membrane, and an icosahedral protein capsid external to the internal membrane [17–20]. The viral capsid is composed mainly by the capsid protein p72 [21]. The structural protein p150 is the product of the proteolysis of the polyprotein p220 from the core shell. Some virions are enveloped and the outer envelope is acquired from the plasma membrane when the virus exits the cell by budding. This membrane is dispensable for infection [21].

Following a complex entry mechanism [22–24], ASFV should traffic the endocytic pathway for a successful infection [25]. Then, the virus undergoes sequential uncoating at the endosomal pathway following subsequent steps [26]. The acidic environment of late endosomes is necessary for the virus to loss its capsid and acidification inhibitors such as Bafilomycin inhibit decapsidation and further infectivity. Also, alterations of cholesterol transport inhibit virion fusion and progression along the endocytic pathway [27]. After fusion of viral internal membrane with the viral membrane, naked cores are released to the cytoplasm to start replication [24]. Early mRNA synthesis begins in the cytoplasm immediately after virus entry and is regulated by enzymes and factors packaged in the virus core. After early protein expression, virus DNA replication starts approximately at 6 hours postinfection (hpi) and viral morphogenesis takes place in cytoplasmatic viral factories [21]. Following an expression pattern similar to poxvirus, intermediate and late viral proteins are synthesized after virus replication. Specifically, major infection proteins p54 and p72 accumulate at the replication site or viral factory [20,28]. After viral morphogenesis, the new progeny is released by budding [21].

There are no reports regarding the effects of proteasome inhibitors on ASFV replication, which encodes a highly homologous gene to the E2 or ubiquitin conjugating (UBC) enzyme (UBCv1) [29]. This ASFV encoded enzyme was shown to be functionally active when expressed in Escherichia coli [29]. The diversity of UBC enzyme functions suggests a variety of possible roles for UBCv1 in either regulating the virus replication cycle or modulating host cell function. It is conceivable that this E2 enzyme could act during the onset of DNA replication, at the switch of early to late expression, or during virus morphogenesis. Alternatively, UBCv1 may target molecules for proteolysis and thus modulate host cell function. Then, we set out to study the ubiquitin-proteasome system in African swine fever infection.

## Materials and methods

### Cells, viruses, and infections

Vero (ATCC CCL-81, Richmond, VA, USA) and Cos-7 (ATCC CRL-1651, Richmond, VA, USA) cell lines were maintained in Dulbecco´s modified Eagle medium (DMEM) containing 100 IU/ml penicillin, 100 g/ml streptomycin, 2 mM GlutaMAX and supplemented with 5% and 10% of heat-inactivated fetal bovine serum (FBS), respectively. FBS was reduced to 2% in the inoculum at the time of viral adsorption and throughout the infection process. Swine alveolar macrophages were isolated by flushing the lung with phosphate-buffered saline (PBS), as described [30], and cultured in RPMI medium with 10% of FBS, 2mM GlutaMax, 50 μM 2-mercaptoethanol, 20mM Hepes and 30 μg/ml gentamicin. We used the cell culture-adapted non-pathogenic ASFV isolate Ba71V which was propagated in Vero cells as previously described [31]. Ba71V was used at a multiplicity of infection (moi) of 1 or 10 pfu/cell as indicated. When synchronization of infection was required, virus adsorption was performed for 90 min at 4°C followed by a cold phosphate-buffered saline (PBS) wash to remove unbound virus, then, cells were rapidly shifted to 37°C with fresh pre-warmed medium. When indicated, ASFV was partially purified by a sucrose cushion (40%) in PBS at 20000 rpm for 50 min at 4°C. For comparison, we used a recombinant vaccinia virus (VV) named vtag2GFP containing the tag2GFP under the control of a strong synthetic VV early/late promoter [32].

### Generation of fluorescent recombinant virus BPP30GFP

An African swine fever virus recombinant expressing the GFP gene fused to the promoter of the early viral p30 protein was constructed. Generation of the insertion vector involved several steps. A fragment of 199 bp corresponding to p30 promoter was obtained from BA71V genome by PCR with specific primers (5′- GCGCGGATCCCCCGGTAACTCCGTGTTTGTAC -3′ and 5′-GCGCGAATTCGGATATATTTAAAATAAAATCC -3′). The plasmid pinsp37-GFP was used to obtain the GFP coding sequence. This plasmid was generated in our lab (S1 Fig) modifying the plasmid pINS72β-gal [33]. In order to generate the vector pinspp30-GFP, previous PCR product and pinsp37-GFP were digested with EcoRI and BamHI and cloned into the 4.7 Kbp fragment resulting from the digestion of plasmid pINS72Gal [34,35] with BamHI. PINSpp30-GFP contains GFP coding sequence under control of p30 promoter and flanked by two fragments of the viral gene K196R that codify for the thymidine kinase (TK).

Cos cells infected with Ba71V at a moi of 1 pfu/cell for 1 h were transfected with pINS-pp30GFP using Lipofectamine2000 (Life Technologies), according to manufacturer’s instructions. The insertion of the pp30-GFP coding sequence into thymidine kinase locus (TK) in Ba71V genome was achieved by homologous recombination. When the cytopathic effect was completed (approximately 72 hpi), the cultures were harvested and sonicated. These transfection-infection mixtures were used to infect monolayers of Cos cells seeded onto 6-well dishes at different dilutions. After 5–6 dpi, the recombinant virus generated was isolated from progeny virus by three rounds of plaque purification on Cos cells. During this time, recombinant virus plaques were screened for EGFP direct fluorescence. Furthermore, growth curves obtained with recombinant virus indicated identical replication behaviour and infectious efficiency when compared to the parental strain. The resulting recombinant virus was named BPP30GFP (S1 Fig).

### Chemicals agents

Proteasome inhibitors MG132 (Calbiochem) and Bortezomib (Santa Cruz Biotechnology) were dissolved in dimethyl sulfoxide (DMSO) at stock concentrations of 10 mM and Lactacystin (Santa Cruz Biotechnology) at 5 mM. MG132 was used at concentrations of 0.1, 0.5 and 1 μM, Lactacystin at 5, 10 and 20 μM and Bortezomib at 0.01, 0.1 and 0.5 μM. Endosomal acidification inhibitor Bafilomycin (Sigma Aldrich) was dissolved in DMSO at a stock concentration of 100 μM and used at 200nM. Working solutions at indicated concentrations were freshly prepared in DMEM 2% FBS. Prior to infection, cells were pretreated for 1 h with MG132, Lactacystin, Bortezomib and Bafilomycin. Drugs were present throughout the course of the experiment unless otherwise specified. In order to determine above indicated working concentrations, we analysed cell viability and cytotoxicity tests under every drug treatment with the CellTiter 96 Non-radioactive Cell Proliferation Assay (Promega), following manufacturer’s instructions. The cytotoxic activity of the organic solvent DMSO was also included. We also confirmed the cell viability of these concentrations by counting the number of cells after each treatment. Vero cells were seeded in 24-well plate and treated with the highest non-toxic concentration for 16h and harvested. We counted them using Trypan Blue staining. Based on these results we selected the optimal non-toxic or working concentrations for the infection assays.

### DAPI staining and cell cycle analysis

We used flow cytometry to study the cell cycle profile of Vero and 293T cells treated with proteasome inhibitors. We used DAPI to dye DNA and determine the proportion of those cells in G1, S and G2 phase. After 16h of treatment cells from a T-25 flask were detached with trypsin, washed with PBS1X and fixed with 2ml of cold ethanol 70%. After washing the cells twice with PBS1X-Triton X100 0.03%, DNA is stained with 500 μl of DAPI solution (DAPI at 1μg/ml in PBS1X-Triton X100 0.03%) at 4 C O/N. Labeled cells were analysed for DAPI staining using FACS Canto II flow cytometer (BD Sciences) and percentages of cells in G1, S or G2 phases were processed using FlowJo software.

### Flow cytometry

Vero cells were pretreated with inhibitors MG132, Lactacystin and Bortezomib at the indicated concentrations, followed by infection with Ba71V at a moi of 1 pfu/cell. Cells were washed with PBS after 90 min of adsorption at 37°C, and incubated with DMEM 2% 16 h. Cells were then harvested by trypsinization, and washed with flow cytometry buffer (PBS, 0.01% sodium azide, and 0.1% bovine serum albumin). Cells were fixed and permeabilized with Perm2 (BD Sciences) for 10 min at room temperature (RT). Detection of ASFV infected cells was performed by incubation with anti-p30 monoclonal antibody (diluted 1:100; kindly given by Dr. Escribano, INIA) or anti-p72 monoclonal antibody (1:1000; clone 1BC11 for immunofluorescence, Ingenasa) for 30 min at 4°C, followed by incubation for 30 min at 4°C with phycoerythrin (PE)-conjugated anti-mouse immunoglobulins (Dako) diluted 1:50. In order to determine the percentage of infected cells per condition, 10,000 cells/time point were scored and analysed in a FACS Canto II flow cytometer (BD Sciences). Infected cell percentages obtained after drug treatments were normalized to control values.

### Confocal microscopy

Vero cells were seeded at 60% confluence onto glass coverslips in 24 well plates prior to infection or drug treatment. Then, cells were washed with PBS and fixed with 4% paraformaldehyde (PFA) for 15 min. After a PBS wash, cells were permeabilized 10 min with 0, 1% Triton X-100 in PBS. Then, coverslips were washed with PBS and incubated for 1 h in 2% bovine serum albumin (BSA, Sigma) diluted in PBS. Slides were then incubated for 1 h in primary antibody diluted in 1% BSA in PBS. The appropriate secondary antibody conjugated to Alexa Fluor-488 or -594 (Life Technologies) was used and cell nuclei detected with TOPRO3 (Molecular Probes). TOPRO3 stain has a very strong binding affinity for dsDNA and due to a carbocyanine-based dye generates far-red fluorescence. Coverslips were mounted on glass slides using ProLong Gold (Life Technologies). Cells were visualized using TCS SPE confocal microscope (Leica) and data analysed using Leica Confocal Software.

Primary antibodies used for immunofluorescence assays included the following: mouse monoclonal antibody to 20S proteasome (Merck Millipore) at 1:50 dilution; rabbit monoclonal antibody for the detection of polyubiquitin chains linked through the Lys48 residue of ubiquitin (Merck Millipore) 1:100, rabbit monoclonal for polyubiquitin chains linked through Lys63 residue of ubiquitin (Merck Millipore) 1:100, anti-ASFV p30 mouse monoclonal antibody at 1:100 (kindly given by Dr. Escribano, INIA), anti-ASFV mouse monoclonal antibodies anti-p72 (clone 1BC11 for immunofluorescence, Ingenasa) at 1:1000 and anti-p150 (clone 17AH2, Ingenasa) at 1:100 dilutions.

### Western blotting

Vero cells seeded in 6-well plates were infected with ASFV in the presence or absence of inhibitors at a moi of 1 pfu/cell. Cells were lysed with RIPA buffer (50mM TrisHCl pH7.4, 1mM EDTA, 1mM EGTA, 100mM NaCl, 1% Triton X100, 0.2% sodium deoxycholate and 0.1% SDS) and electrophoresed in sodium dodecyl sulfate polyacrylamide gels and transferred onto nitrocellulose membrane (GE Healthcare). The membrane was incubated in blocking buffer (5% BSA in PBS) for 1 hour, prior to overnight incubation with primary antibodies diluted in 2% BSA in PBS. Antibodies were detected with horseradish peroxidase (HRP)-conjugated secondary antibodies and bands obtained were detected with the ECL system (Amersham) using a Chemidoc XRS imaging system (BioRad). Band densitometry was performed with Image Lab software (BioRad) and data obtained were normalized to control values. Primary antibodies used for western blot included: anti-ASFV p30 mouse monoclonal antibody, at 1:500, anti-ASFV p72 mouse monoclonal antibody (clone 1BC11, Ingenasa) at 1:1000, and mouse monoclonal to tubulin (Sigma-Aldrich) at 1:2000, used as load control.

### Quantitative real time PCR

DNA from Vero cells and alveolar macrophages treated with the indicated concentrations of the proteasome inhibitors and infected with ASFV at a moi of 1 pfu/cell for 16 hpi were purified using the DNAeasy blood and tissue kit (Qiagen). Untreated- and DMSO-treated cells were used as a control. DNA concentration was measured using a Nanodrop spectrophotometer. The qPCR assay used fluorescent hybridation probes to amplify a region of the p72 viral gene, as described previously [36]. The amplification mixture was 300 ng of DNA template added to a final reaction mixture of 20 μl containing 50 pmol sense primers, 50 pmol anti-sense primer, 5 pmol of probe and 10 μl of Premix Ex Taq (2X) (Takara). Positive amplification controls were DNA purified from ASFV virions at different concentrations as standards. Each sample was included in triplicates and values were normalized to standard positive controls. Reactions were performed using the ABI 7500 Fast Real-Time PCR System (Applied Biosystems) with the following parameters: 94°C 10 min and 45 cycles of 94°C for 10 sec and 58°C for 60 sec.

### Fluorometric assay

The activity of the proteasome was measured using the Proteasome Activity Assay Kit (Merck Millipore) at a range of times post-infection in Vero cells seeded in 24 well-plates and infected with ASFV at a multiplicity of 1 pfu/cell. The assay was based on the detection of the fluorophore 7-Amino-4-methylcoumarin (AMC) after cleavage from the labelled substrate LLVY-AMC. Results were analysed by Tecan GENios fluorometer and XFlour 4 software (Tecan Switzerland) after 60 minutes of reaction, following the manufacturer´s instructions.

### Virus titration

Vero cells were infected after drug treatment with Ba71V, VV or BPP30GFP at a moi of 1 pfu/ml. After 24 h of infection, total viruses from cell lysates and supernatants were collected and titrated by plaque assay in triplicate samples on monolayers of Vero cells. Cells were infected with 10-fold serial dilutions from samples and viral adsorption lasted 90 min in 2% FBS at 37°C. The viral inoculum was then removed and a 1:1 of 2% low-melting-point agarose and complete 2X EMEM was added. Plaque visualization was possible at 10 days after staining with violet crystal. When using the recombinant BPP30GFP green fluorescent plaques were observed 4 days after infection.

### Statistical analysis

The experimental data were analysed by one-way ANOVA by Graph Pad Prism 5 software. For multiple comparisons, Bonferroni´s correction was applied. Values were expressed in graph bars as mean±SD of at least three independent experiments unless otherwise noted. A *p* value <0.05 was considered statistically significant.

## Results

### Proteasome inhibitors decreased ASFV infection

**Fig 1:**
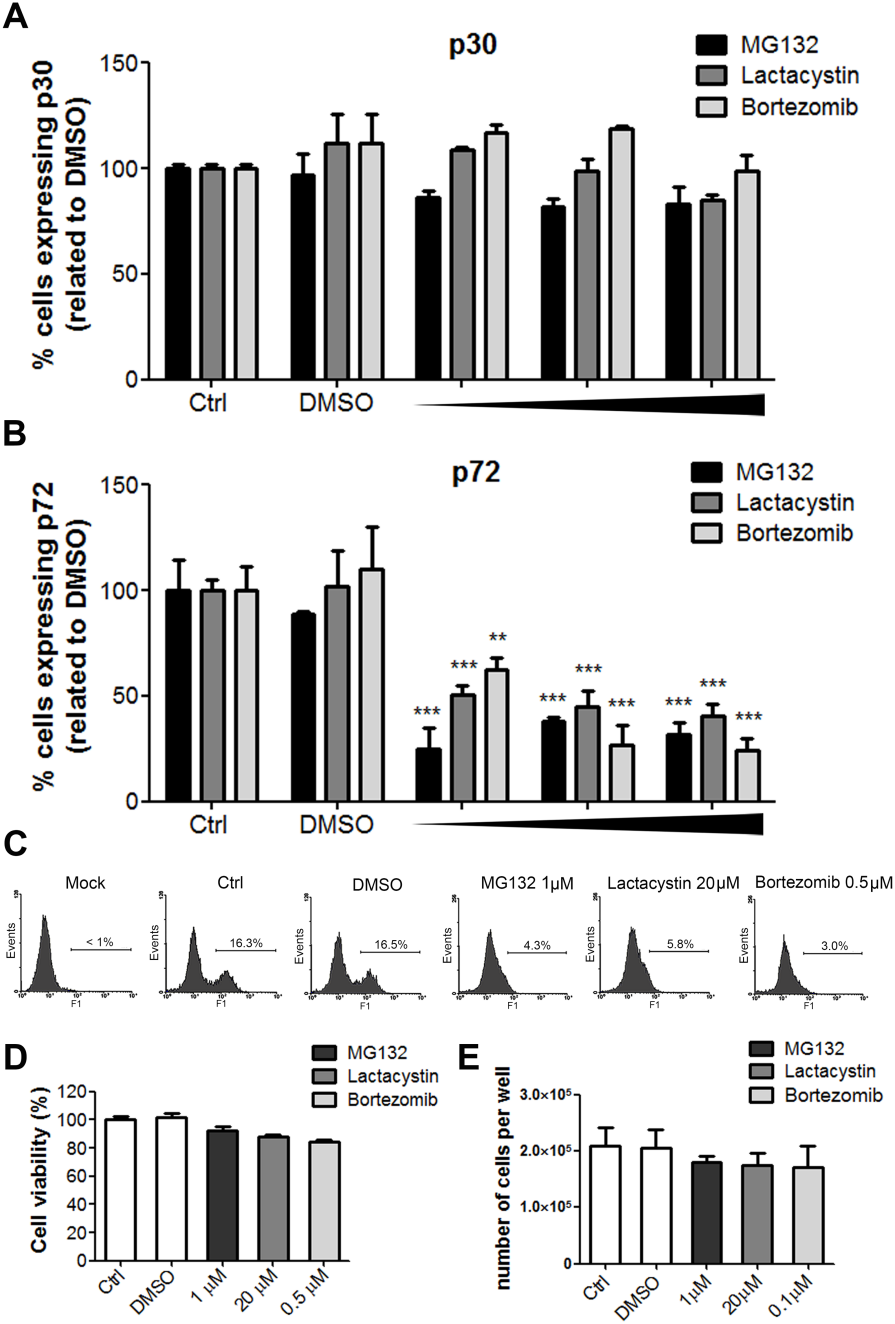
Effect of proteasome inhibitors on ASFV infection. Percentages of ASFV Ba71V-infected cells analysed by flow cytometry at 16 hpi using monoclonal antibodies against early p30 (A) and late p72 (B) viral proteins. Vero cells treated with increasing doses of MG132 (0.1, 0.5 and 1 μM), Lactacystin (5, 10 and 20 μM) and Bortezomib (0.01, 0.1 and 0.5 μM) 1 h prior to infection or left untreated. Data normalised to controls were expressed as mean±SD of three independent experiments and compared to DMSO. Significant differences were marked with asterisks as indicated (**p<0.01; ***p<0.001). (C) Representative flow cytometry profiles of the % of cells expressing the late protein p72. (D) Cytotoxicity assay of inhibitors MG132 (1 μM), Lactacystin (20 μM) and Bortezomib (0.5 μM) used to select the non-toxic working concentrations. (E) Cell viability analysis by counting the number of cells with Trypan Blue stain. Cells were treated with the inhibitor MG132 (1 μM), Lactacystin (20 μM) and Bortezomib (0.5 μM) for 16h.

First, we analysed the effect of proteasome inhibition at the final stage of the ubiquitin-proteasome pathway. We used MG132, widely used as a reversible and cell permeable proteasome inhibitor [37], Lactacystin, an irreversible proteasome inhibitor [38] and the 26S subunit proteasome inhibitor Bortezomib [39–41]. To determine whether proteasome inhibition could influence ASFV infection, we treated cells for 1 h with several concentrations of MG132, Lactacystin or Bortezomib and then infected with ASFV (Fig 1). We calculated the non-toxic highest concentration of these inhibitors without an effect in cell viability (Fig 1D and 1E). Also, cell cycle analysis showed no changes in S-phase in the time postinfection analysed (16 hpi; S2 Fig). After 16 hpi, ASFV infectivity was analysed by flow cytometry by early p30 and late p72 protein expression using monoclonal antibodies against these viral proteins. While the percentage of cells expressing p30 was not affected at any drug concentration (Fig 1A), the percentage of cells expressing late protein p72 decreased in cells treated with proteasome inhibitors (Fig 1B and 1C). These results indicated that proteasome inhibition did not affect early gene expression. However, proteasome inhibitors reduced the number of infected cells expressing late protein p72, inhibiting infectivity up to 76% in a dose dependent manner (Fig 1B–1D).

### MG132 treatment reduced ASFV replication, late viral protein synthesis and virus production

**Fig 2:**
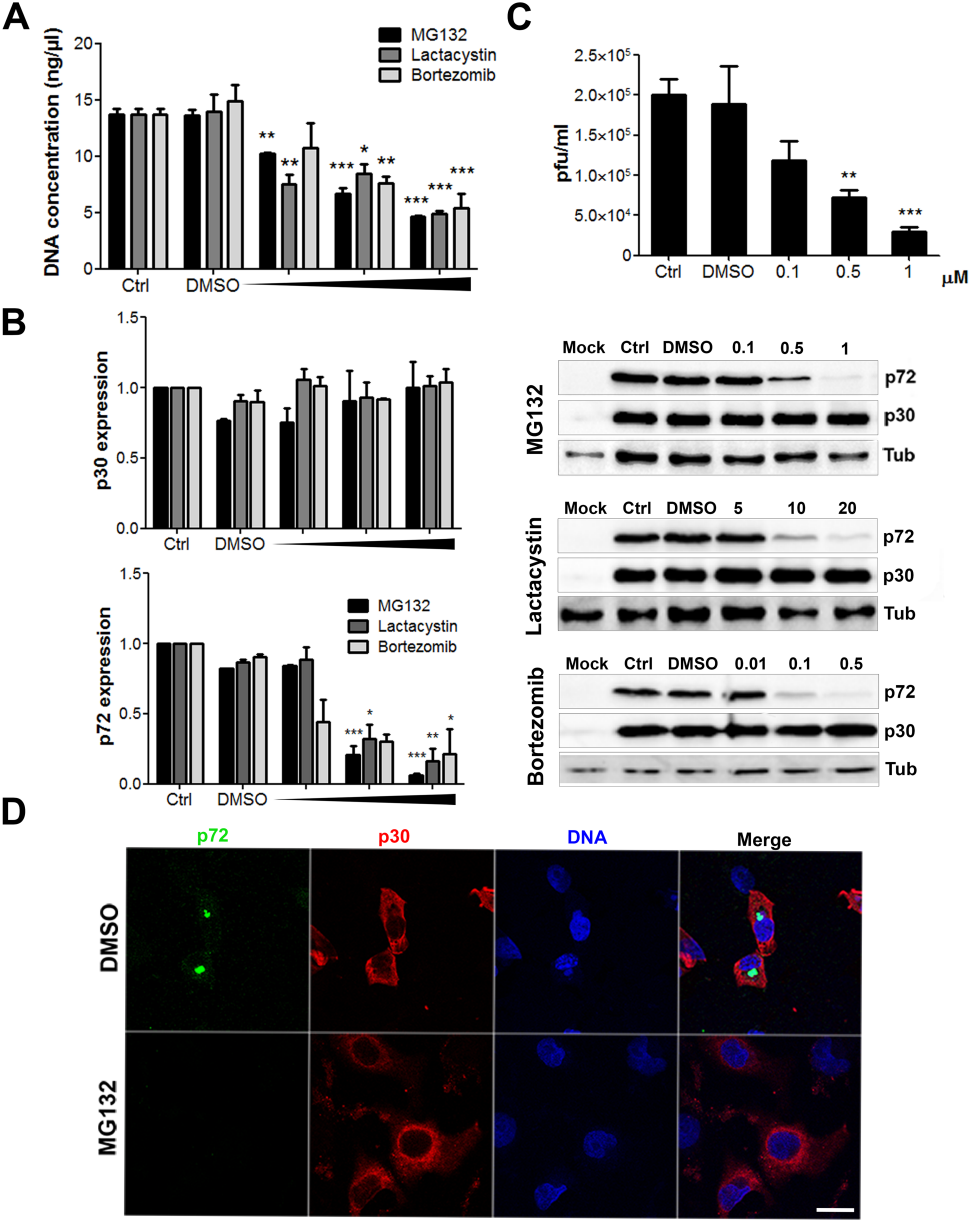
Analysis of proteasome inhibition on several infection parameters. (A) Quantitation of ASFV viral DNA at 16 hpi in Vero cells pretreated 1 h with several concentrations of MG132, Lactacystin and Bortezomib. Data were compared to DMSO. Significant differences are marked with asterisks as indicated (*p<0.05; **p<0.01; ***p<0.001). (B) Representative western blot images of early p30 and late p72 expression in cells pretreated with MG132, Lactacystin and Bortezomib and infected with ASFV at 16 hpi. A sample WB image for MG132. Lactacystin and Bortezomib (from top to bottom) is shown. Quantification of the bands was corrected with tubulin data, normalised to controls values and compared to DMSO. Graphics depict mean±SD of densitometry values from three independent experiments. (C) Virus titration by plaque assay of Vero cells infected with recombinant virus BPP30GFP at a moi of 1 pfu/cell for 24 hpi in the presence of the inhibitor. (D) Representative confocal micrographs of Vero cells treated with 1 μM MG132 and infected with ASFV for 16h. Infected cells were labelled for viral protein p72 (green), which labels the viral factories, or early viral protein p30 (red), which shows a characteristic diffuse cytoplasmic staining. Bar = 10μm.

Then, we further analysed how the viral cycle was affected by proteasome inhibition. ASFV gene expression follows a cascade mechanism similar to that described for poxvirus [42]. Immediate-early and early genes are expressed before DNA replication begins. DNA replication is due to the action of several enzymes that are packed in the viral core and are involved in the viral transcription. In contrast, the expression of intermediate and late genes is delayed and dependent on *de novo* synthesis of specific transcription factors and viral DNA. Then, considering the decrease in the number of cells expressing p72 found in infected cells treated with these proteasome inhibitors, we further analysed the status of ASFV viral DNA replication at the same conditions. Total DNA from infected cells treated with several concentrations of MG132, Lactacystin and Bortezomib was isolated and analysed by qPCR. We detected that the presence of the proteasome inhibitors produced a 3-fold inhibition of viral DNA replication (Fig 2A). In addition, we studied other altered infection parameters. Late protein expression following MG132 treatment was 10-fold reduced at the highest concentration of drug in infected cells, while the production of early protein p30 was not significantly modified (Fig 2B). Using increasing concentrations of Lactacystin and Bortezomib we observed a 5- and 4-fold reduction respectively in late protein expression while p30 remained unaltered (Fig 2B).

Furthermore, we generated a new tool, an ASF recombinant virus expressing the GFP gene fused to the promoter of the early viral p30 protein. The virus BPP30GFP was generated as described in Material and Methods and was used for titration. Our above observation of impaired DNA replication under proteasome inhibitors was further supported by a 6-fold decrease in green plaques production using recombinant BPP30GFP in cells pretreated with the proteasome inhibitor (Fig 2C). Then, we analysed viral factory (VF) formation under MG132 proteasome inhibition. We studied the morphology and numbers of VF in cells infected for 16 h in the presence and absence of MG132 using antibodies against major viral capsid protein p72 and ds-DNA staining Topro3 by confocal microscopy. Infected untreated cells presented characteristic p72-positive perinuclear viral factories that were absent upon MG132 treatment (Fig 2D).

**Fig 3:**
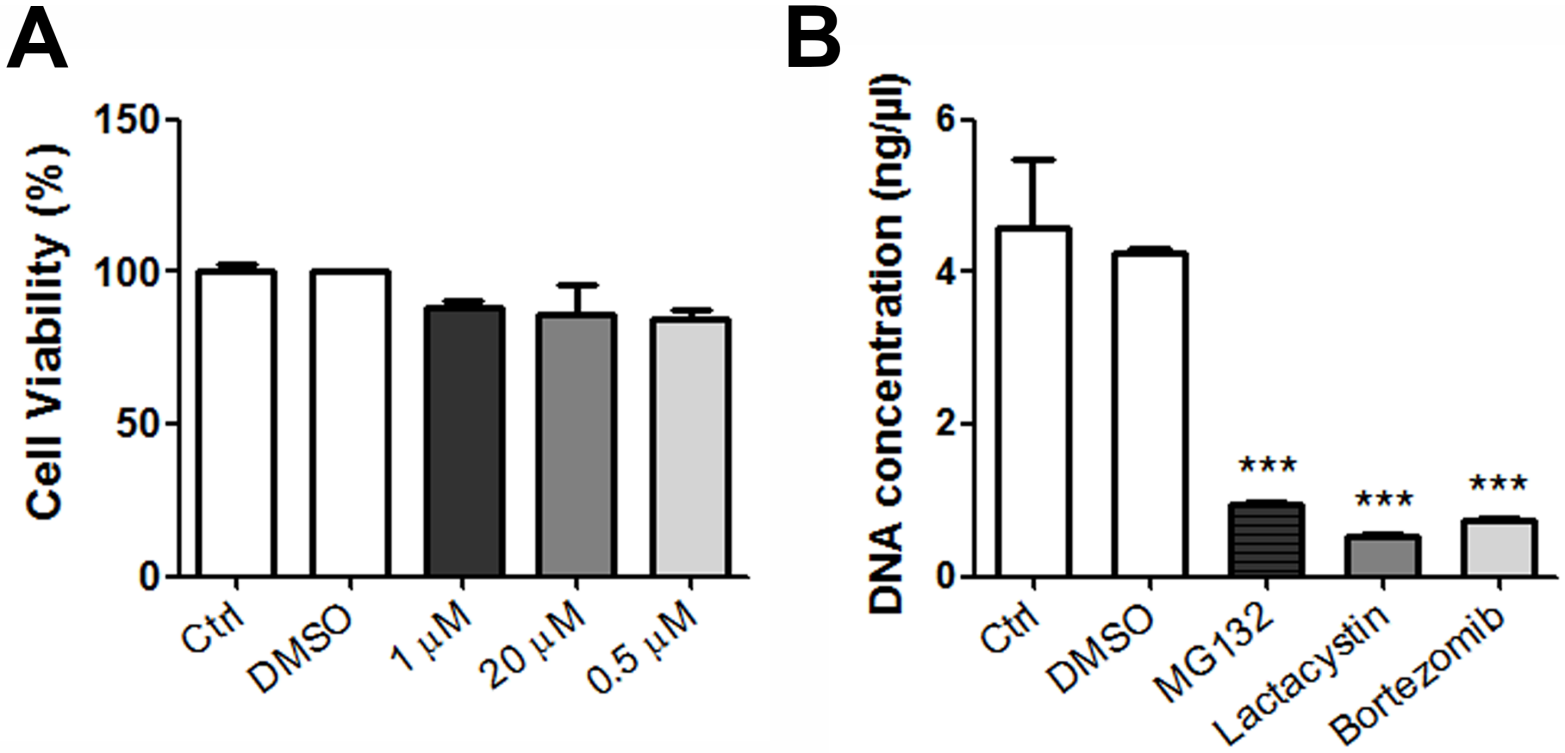
Proteasome inhibitors affected ASFV replication in porcine macrophages. (A) A cytotoxicity assay of inhibitors MG132 (1 μM), Lactacystin (20 μM) and Bortezomib (0.5 μM) was used to select the non-toxic working concentrations. (B) Quantitation of ASFV viral DNA at 16 hpi in macrophages pretreated 1 h with 1μM MG132, 20μM Lactacystin and 0.05μM Bortezomib. Data were compared to DMSO. Significant differences are marked with asterisks as indicated (*p<0.05; **p<0.01; ***p<0.001).

Next, we decided to study the effect of proteasome inhibitors in porcine macrophages, the main target for ASFV [43]. We used the highest non-toxic concentration of each drug (Fig 3A) to study the impact of the inhibition of the proteasome in viral replication. We extracted the total DNA from infected macrophages treated with 1μM MG132, 20 μM Lactacystin and 0.5μM Bortezomib and analysed by qPCR. The treatment of porcine macrophages with proteasome inhibitors implied at least a 4-fold reduction in ASFV replication (Fig 3B).

### MG132 acts at an early step of ASFV infection

**Fig 4:**
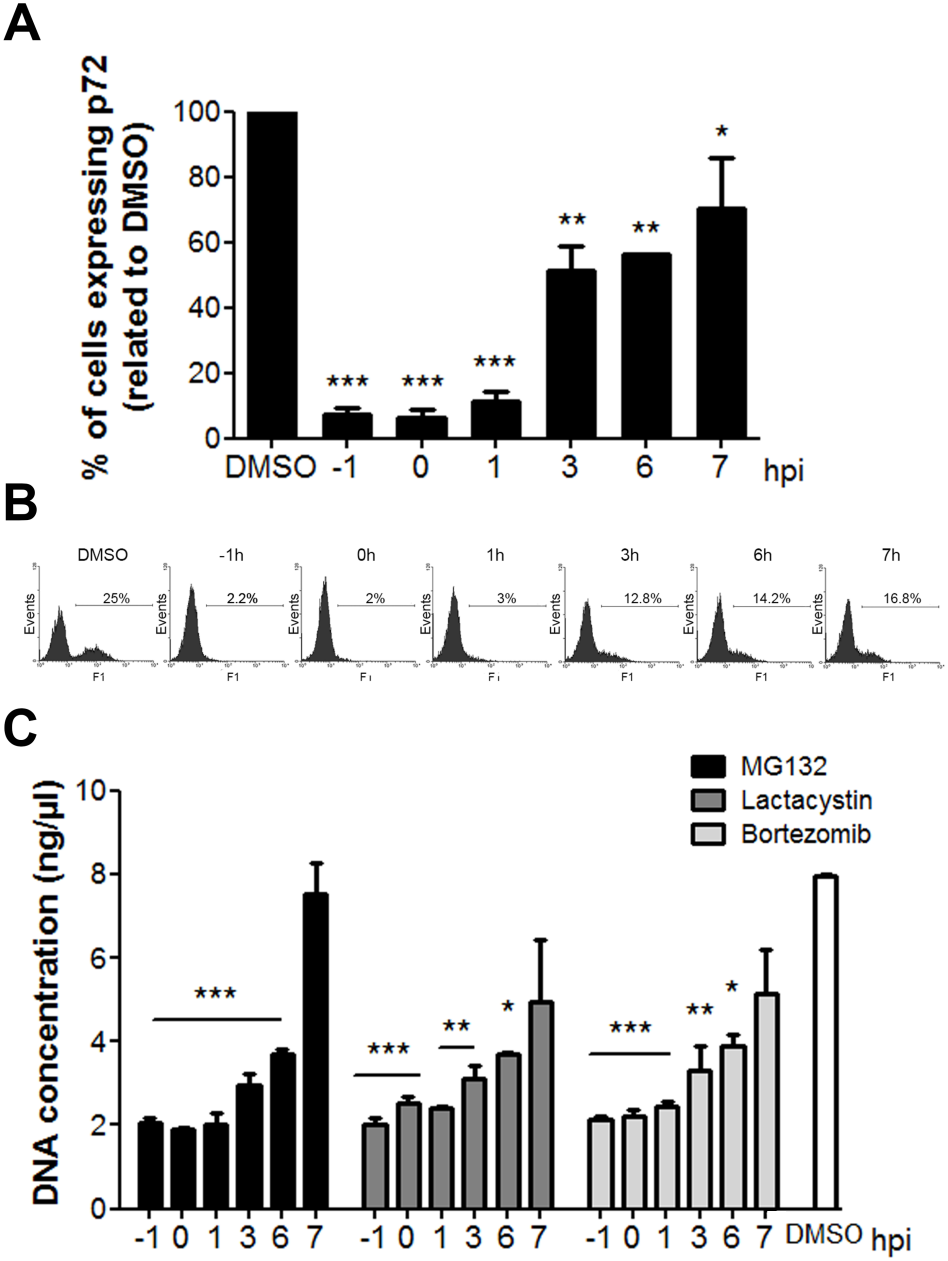
Proteasome function is required at an early stage of infection. (A) Time-course of ASFV infectivity under inhibition with MG132. Inhibitor was added either 1 h before (-1 hpi), at the time of infection (0 hpi) or at 1 to 7 hpi. Infectivity was measured as the percentage of cells expressing the late protein p72 at 16 hpi by flow cytometry. (B) Representative flow cytometry profiles of each time of treatment. (C) Quantification of viral DNA replication in Vero cells treated with MG132, Lactacystin or Bortezomib 1 h prior or 0 to 7 h after infection. Mean±SD correspond to three independent experiments. Differences are marked with asterisks as indicated (*p<0.05; **p<0.01; ***p<0.001).

In order to investigate whether MG132 was specifically blocking virus entry or subsequent events at later stages of the infection cycle, we analysed the percentage of cells expressing p72 at 16 hpi by flow cytometry adding the inhibitor at different hpi. The number of infected cells was significantly reduced when MG132 was added 1 h before infection but was moderate when inhibitor was added later, at 3 hpi (Fig 4A and 4B). This result indicates that proteasome activity is required during early stages of infection. Similarly, we further studied the effect of MG132, Lactacystin and Bortezomib on viral replication at these times. Cells were pretreated for 1 h or treated at several postinfection times with 1 μM MG132, 20 μM Lactacystin or 0.5 μM Bortezomib. Total DNA from infected cells treated with the inhibitors was isolated and analysed by qPCR. We found that inhibition of the proteasome with MG132 produced a 4-fold inhibition when added prior, at the time of infection or at the first hpi (Fig 4C). This inhibition decreased moderately when treatment was started at 3 or 6 hpi and no changes in DNA replication were observed when the drug was added later than this time point. Lactacystin and Bortezomib caused 2.2-fold inhibition when added at any time during the first hpi but inhibition was also moderate after this time point. With these data we could conclude that the timing when an active proteasome is needed for ASFV infection is between 0–6 hpi. The earlier the treatment was applied, the more significant effect was found in infection (Fig 4C).

### MG132 prevents DNA uncoating in ASFV

**Fig 5:**
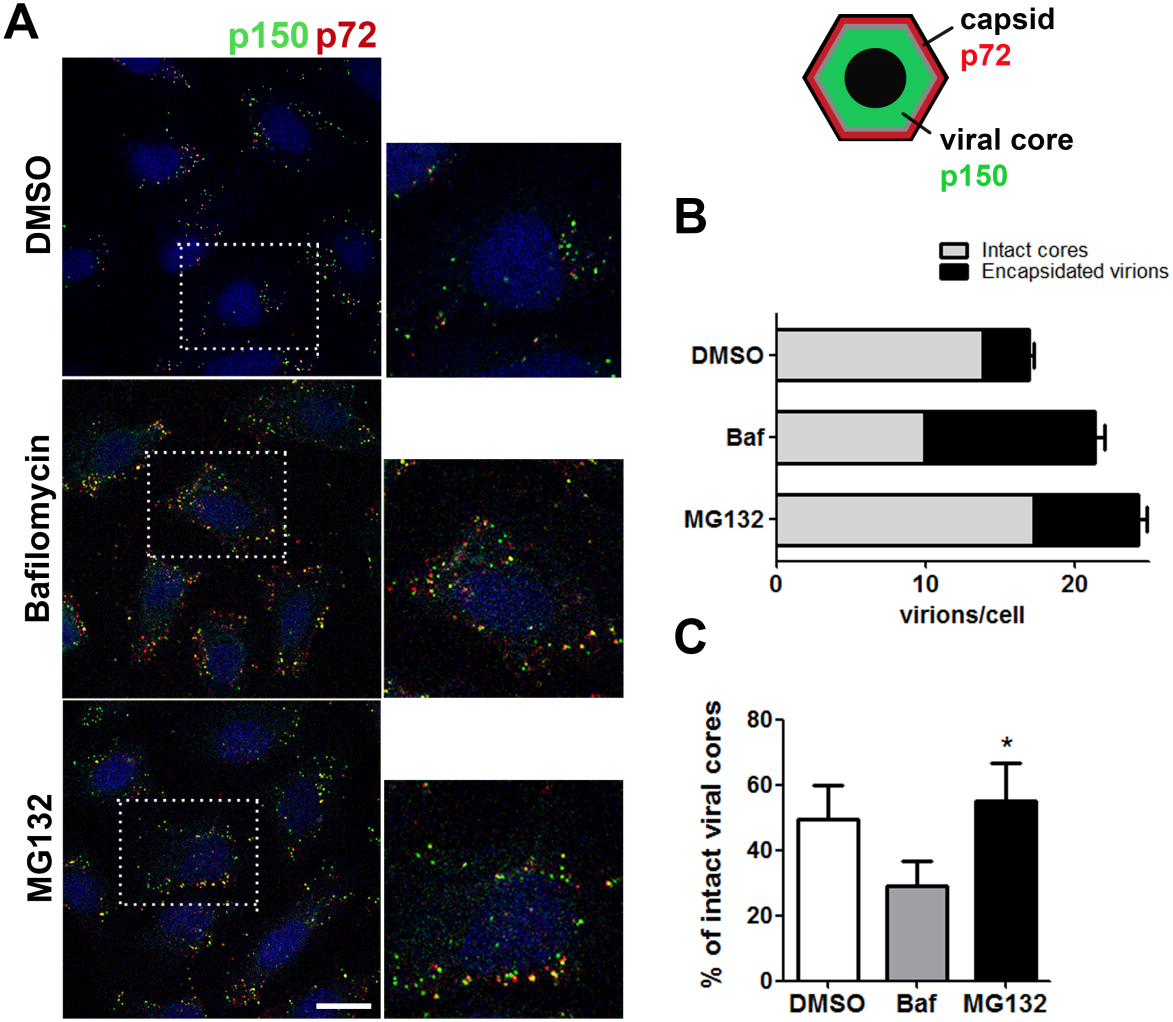
Proteasome inhibition decreased core breakdown of ASF viral particles. (A) Schematics show the disposition of virion layers. The outer capsid composed by ASFV p72 major capsid protein is represented in red colour and the inner core with the viral core protein p150 in green colour. Encapsidated virions would double label to both proteins in yellow while uncoated virions lose capsid staining and would single label in green. Empty virions positive for capsid protein p72 yielded a red signal. Representative confocal microscopy images of ASFV infected cells labelled for viral major capsid protein p72 (red) and inner core protein p150 (green). Cells were pretreated with 1 μM MG132, 200 nM Bafilomycin and infected for 3 hpi at a moi of 10 pfu/cell. Bar = 10μm (B) Number of intact cores (green) and encapsidated virions (yellow) per cells in each condition 3hpi. (C) Graphical representation showing the percentages of uncoated viral cores in cells treated with DMSO, MG132 and Baf normalized to the total number of virions counted in 50 cells per condition.

We considered the possibility that viral DNA replication failed to occur because the absence of the proteasome function could prevent complete virion uncoating prior to replication. Hence, ASFV genome would remain inside the virus core impairing access of the newly synthesized viral replication proteins to start DNA replication. To test this model, we performed a decapsidation assay. Vero cells were pretreated 1 h with DMSO, Baf or MG132 and then infected at a moi of 10 pfu/cell. After viral synchronization at 4°C for 90 minutes, infection was allowed to proceed for 3 h. Then, cells were washed with cold PBS 1X and treated with 0.05% trypsin-EDTA for 10 minutes at 37°C to remove the membrane-bound virus. We used Bafilomycin (Baf), a specific inhibitor of vacuolar type H+-ATPase and endosomal acidification, as a control of decapsidation inhibition. Previous studies of ASFV entry demonstrated that internalization of virus particles required a specific temperature and the acid pH of the late endosome [25,44–46]. The decapsidation assay was conducted by quantifying the number of fully encapsidated particles, double labelled for viral capsid p72 and core p150, and the number of decapsidated virions or intact cores with single staining for p150 (Fig 5A and 5B). We counted cores remaining intact (single label) at 3 hpi in 50 cells per condition and treatment. Counts were normalised to the total virion number (double plus single label; Fig 5C). At 3 hpi, virions go through uncoating and most progress towards replication (in DMSO conditions). Contrarily, if uncoating were affected, intact viral cores would accumulate. Intact viral core rates were depicted in the graph and showed a decrease in core breakdown under proteasome inhibition. In cells treated with MG132, the percentage of intact viral cores increased significantly and to a higher extent than in the Baf uncoating-inhibition control where encapsidated virions predominated. Altogether with above data, these findings could indicate a role for the proteasome in viral core degradation.

### Proteasome activity increases at late infection

**Fig 6:**
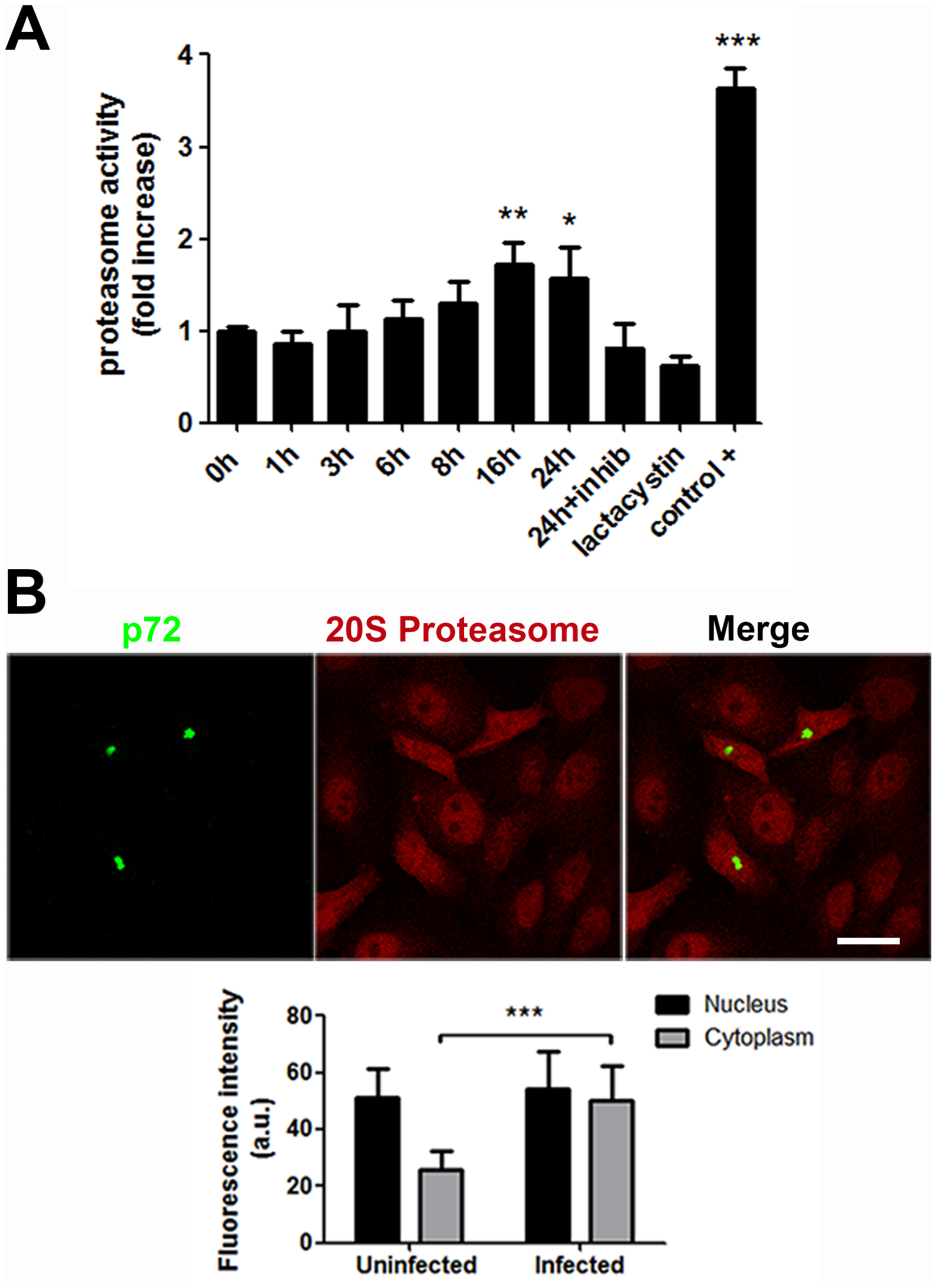
Proteasome activity increase at later times of the infection. (A) Proteolytic activity evaluation at several times after ASFV infection by a proteasome activity assay. Fold changes at several times were compared with uninfected cell values. Also, a proteasome activity inhibitor (Lactacystin) and a very strong proteasome activity inducer were used as controls. (B) Representative confocal images of the distribution of 20S proteasome protein in uninfected or infected cells at 16 hpi. Viral factories (VF) stained with late ASFV protein p72 (green) and 20S proteasome protein (red). Bar = 10μm. Graph represents the fluorescence intensity of 20S proteasome in uninfected and infected cells measured using LAS Application quantification tool and a Leica TCS SPE confocal microscopy. Significant differences are marked with asterisks as indicated (*p<0.05; **p<0.01; ***p<0.001).

Given the relevance of the proteasome for ASFV infection and several other virus models, we next analysed the activity of the proteasome along ASFV infection. A fluorometric assay was performed to measure the proteolytic activity in control or ASFV infected cells harvested at several time points (moi of 1 pfu/cell). Proteasome activity increased significantly later after infection (16 and 24 hpi) compared to uninfected cells. No significant changes were found at early times (1–8 hpi) as we can observe in Fig 6A. We used Lactacystin as a negative control of proteasome activity and also a positive control, both provided by the commercial assay kit (see Materials and methods). This positive control was a very strong proteasome activity inducer and yielded 4-fold activity than uninfected control cells.

Then, we studied the location of the proteasome protein by confocal microscopy in infected cells at 16 hpi. In infected cells proteasome expression shifted from the nucleus (in controls) to the cytoplasm in infected cells recognized as such by p72 staining of the viral factory (Fig 6B). Fluorescence intensity quantification of confocal images from more than 10 low power fields showed that proteasomes were recruited to the cytosol in infected cells. Infected cells showed proteasome staining abundantly distributed throughout the cytosol and nucleus. However, uninfected cells showed a significantly lower expression of the protein in the cytoplasm (Fig 6C).

### Ubiquitination in ASFV infection

**Fig 7:**
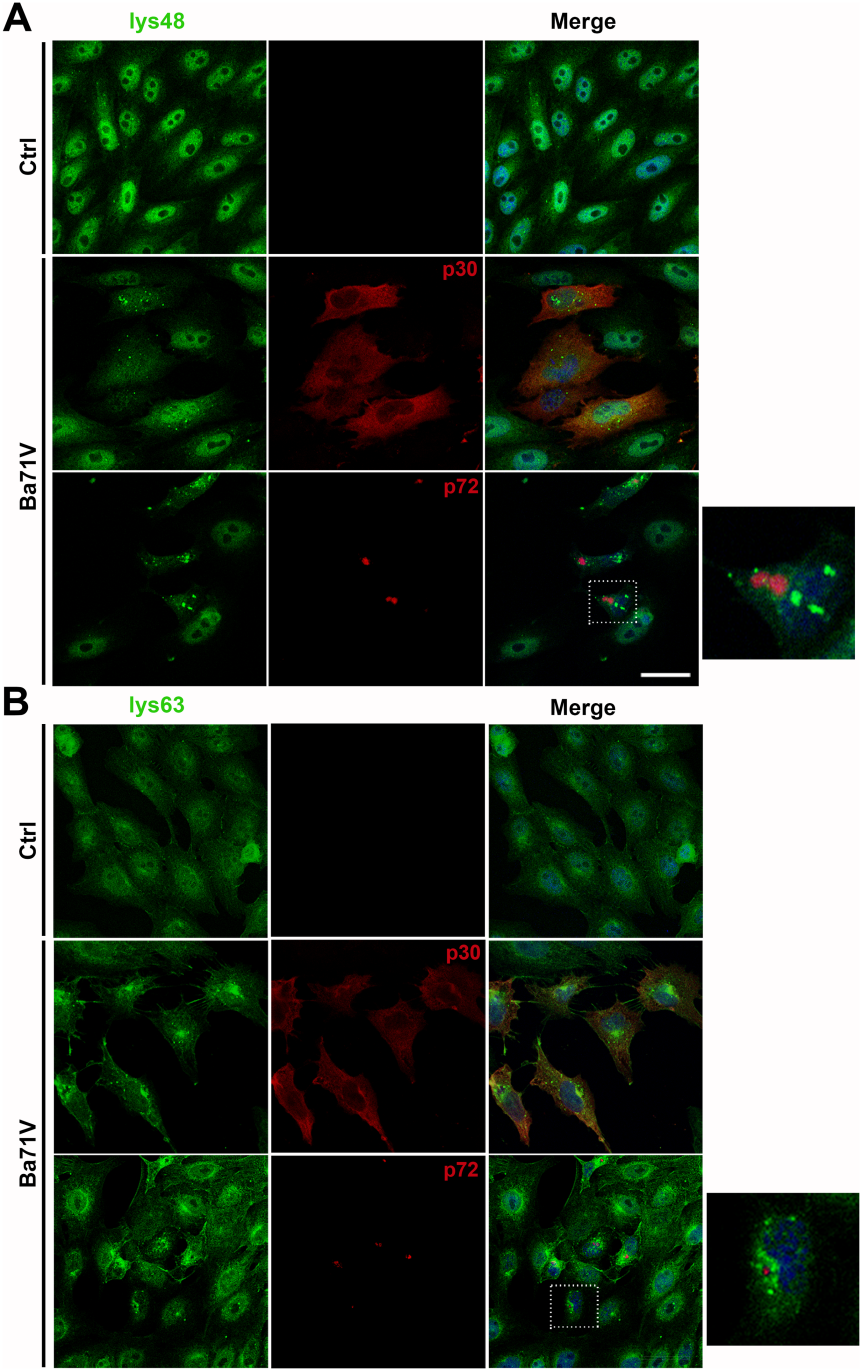
Pattern of poly- and monoubiquitination in ASFV infection. (A) Lys48-UB (green) distribution in control and Ba71V ASFV-infected cells (red) at 16 hpi. In control cells, it was found in the nucleus while it distributed to the cytoplasm in infected cells. Staining for viral p30 was characteristically cytoplasmic while viral p72 accumulated at the viral replication site (red). A high magnification of the merged squared area is shown on the right hand side of the figure. (B) Lys63-UB accumulated around the viral replication sites or viral factories (VF) in infected cells compared to the dispersed distribution controls. Lys63-UB is shown in green and viral proteins in red. Higher magnification of the merged square area is shown on the right. Bar = 10μm.

Finally, we studied the distribution of ubiquitin upon infection. We used antibodies targeted against polyubiquitin chains linked through the Lys 48 (Lys48-UB) or the Lys 63 residues of ubiquitin (Lys63-UB) to study their distribution in control or infected cells (moi of 1 pfu/cell) at 16 hpi (Fig 7). Polyubiquitin chains connected through Lys48-UB are commonly related to proteins degradation by the 26S proteasome. On the other hand, polyubiquitin chains linked through Lys63-UB have non-proteolytic functions associated with signal transduction [47]. We observed that Lys48-UB, which imply a degradative signal, were clearly located in the nucleus in control cells. However, in infected cells, labelled with p30 or p72 (red), Lys48-UB distribution shifted partially to the cytoplasm acquiring a dotted appearance and occasionally labelled inside viral factories (Fig 7A). The regulatory Lys63-UB was found in the nucleus and in the cytoplasm in control cells (Fig 7B). When cells were infected Lys63-UB was recruited and accumulated adjacent to the VF (Fig 7B, zoom).

## Discussion

The UPS plays an important role in the degradation of intracellular proteins and regulates the cellular signalling pathway [48]. Many viruses have manipulated this system to their own advantage [40,49–51]. In the present study, we found that proteasome inhibition impacts ASF infection leading to infectivity reduction, as determined by the decrease in the number of cells expressing late protein p72. However, we excluded that proteasome inhibitor MG132 could directly affect viral entry and/or early gene expression. To ensure that the inhibitory effect of MG132 was due to its suppression of proteasome activity, we tested other proteasome inhibitors such as Lactacystin [39] and Bortezomib [41,52,53]. Some of these inhibitors, as Bortezomib, are currently used for the treatment of multiple myeloma and mantle cell lymphoma [54] and its inhibitory effects in viral infections enabled drug repositioning as antiviral. In fact, several viral infections are sensitive to proteasome inhibitors, among these, herpesvirus [55], vaccinia [56], influenza [57], human immunodeficiency [58], cytomegalovirus [59], hepatitis B [60] and dengue virus [61].

Our data indicate that viral DNA replication was affected by proteasome inhibition. We observed that different proteasome inhibitors decreased of the number of viral DNA copies, reduced viral late protein synthesis, and also total viral production. These results are similar to those described for closely related viruses when the proteasome is inhibited [38,40,59]. Vaccinia virus (VV) requires proteasome activity for the complete uncoating of the viral particle [56,62]. The mechanism by which the proteasome mediates viral uncoating occurs is not completely clear. Apparently, the proteasome recognizes as substrates one or more viral proteins, hence the core particle is destroyed and the viral DNA genome is released to the cytoplasm.

UBCv1 is expressed throughout ASFV infection and accumulates late in infection. The enzyme is also present in purified extracellular virions. The presence of UBCv1 in virions and its accumulation late during infection are consistent with a very early role in infection, immediately following virus entry and before viral transcription. A viral core protein, polyprotein pp62 could be a possible substrate for UBCv in vitro [63]. Polyprotein pp62 is posttranslationally processed to give rise to two major structural proteins of 35 kDa (p35) and 15 kDa (p15) [64]. Monoubiquitinated conjugates are poor substrates for ubiquitin-dependent degradation. But it is also possible that monoubiquitinated structural proteins could act upon virus entry as primers for the formation of a polyubiquitin chain. This could target multiubiquitinated proteins for degradation hence participating in virus uncoating and viral degradation. Then, a mechanism should coexist to prevent its degradation during the previous replication cycle. Alternatively, ubiquitination may play a role in virus morphogenesis late during infection.

According to our results, the impairment of ASFV genome replication by proteasome inhibitors could be consistent with an interference with the complete uncoating of the core, which would eventually prevent access of the replication machinery and subsequent replication. Our findings indicate that inhibition of proteasome function causes arrest at an early stage of ASFV infection, which is also supported by the observations of a statistically significant accumulation of complete viral cores retained in the cytoplasm by confocal microscopy. Then, uncoating inhibition could underlie ASFV replication reduction as it does in VV infection [56].

A possible role of UBCv1 protein in regulating a host nuclear function has been reported [29,65]. However, virus replication sites contain ubiquitinated proteins and also, UBCv1 has been shown to ubiquitinate the viral core polyprotein pp62 or its products *in vitro* [63]. Consequently, the ASFV ubiquitin-conjugating enzyme could exert multiple roles in the infective cycle.

We further showed that ASFV infection has a small effect on the proteasome activity at early times even considering the important role that this system appears to play at these times. It is conceivable that a basal proteasome activity is sufficient for early infection while the saturation of the system in the presence of a high amount of viral proteins requires an increase in activity detected at late infection.

We also analysed the differential ubiquitination patterns of Lys48- and Lys63-linked polyubiquitin chains. Viral factories revealed strong peripheral deposition of Lys63-UB, rather than Lys48-UB, which is typically associated with proteasomal degradation. Lys63-UB chains connect components of NFκB signalling in a highly regulated manner, and genetic evidence indicates a role for Lys63-UB in stress response and DNA repair. The complexity of the interactions carried out by E2 enzymes contradicts their early image as simple carriers of activated ubiquitin. Indeed, it is now evident that E2 conjugating enzymes are able to determine the linkage specificity and length of ubiquitin chains and can influence the processivity of chain formation [66].

Our results indicate that inhibition of the proteasome function causes an arrest at an early stage of ASFV entry into Vero cells. Our future work will be focused on identifying the viral core components that are ubiquitinated as well as the responsible E3 ubiquitin ligase(s). In addition, we should analyse the potential cellular compartments where the viral particle could be arrested by the inhibition of the UPS. This study has demonstrated that the UPS plays an important role at early ASFV infection at a stage comprised between the entry of the virus through the plasma membrane and the replication of the viral genome.

## Supporting information

S1 Fig Generation of fluorescent recombinant virus BPP30 GFP. (A) PINspp30-GFP vector contains GFP coding sequence under control of p30 promoter and flanked by two fragments of the viral gene TK. (B) and (C) Time course of protein expression. (C) Fluorescence in cells infected with the recombinant virus.
(TIF)

S2 Fig Cell cycle analysis by FACS of cells treated with proteasome inhibitors. (A) DNA histograms of cell cycle profile and cell cycle phase quantification of Vero cells treated with 1 μM MG132, 20 μM Lactacystin and 0.5 μM Bortezomib for 16h and subjected to cell cycle analysis by flow cytometry with DAPI staining. (B) DNA histograms and cell cycle phase quantification of HEK293T cells treated with 1 μM MG132, 20 μM Lactacystin and 0.5 μM Bortezomib.
(TIF)
