# Supplementary figures and images for: The ubiquitin-proteasome system is required for African swine fever replication
Source: PLoS One. 2017 Dec 15;12(12):e0189741. doi: 10.1371/journal.pone.0189741 (PMC5731689; doi:10.1371/journal.pone.0189741)

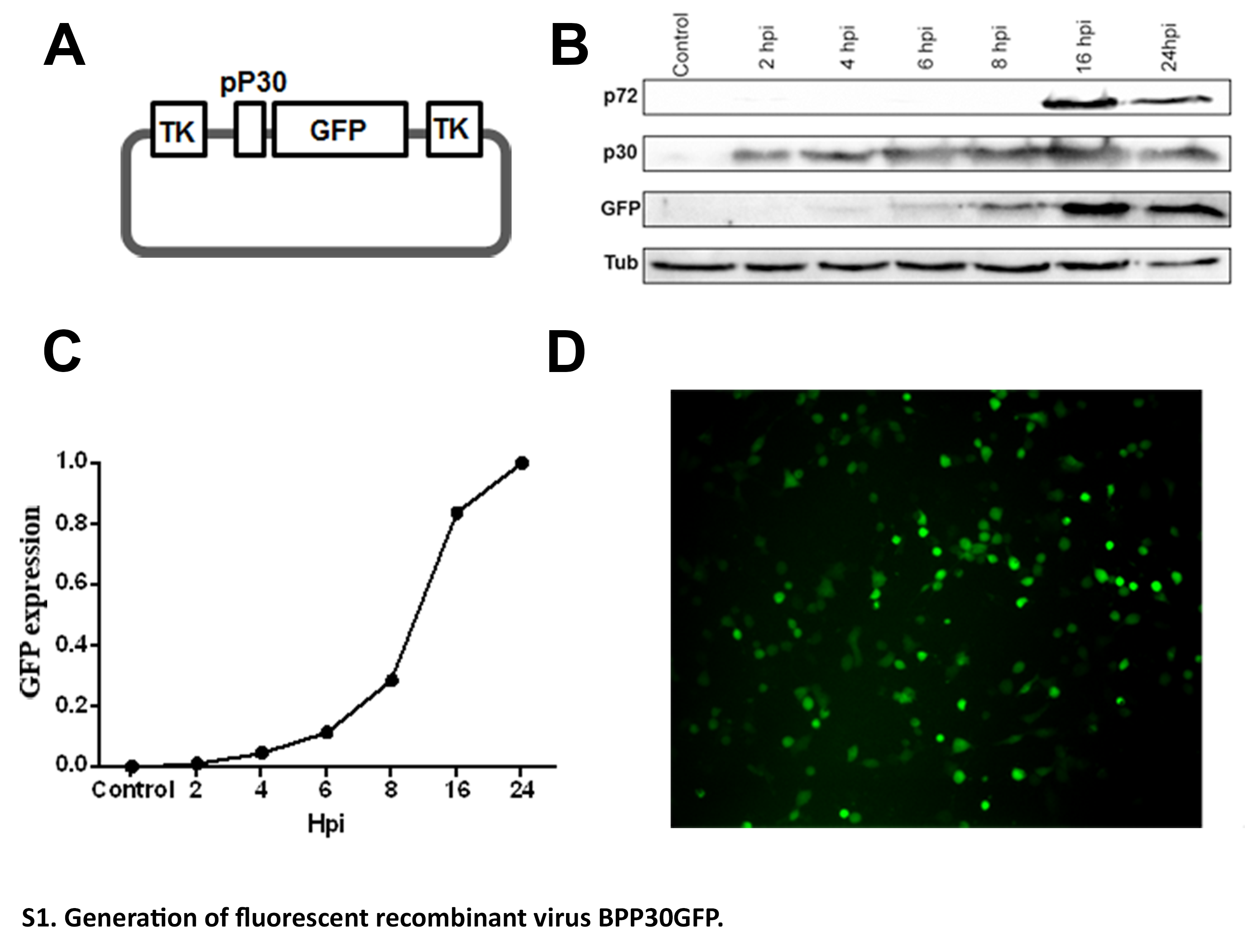

Supplement: S1 Fig — (A) PINspp30-GFP vector contains GFP coding sequence under control of p30 promoter and flanked by two fragments of the viral gene TK. (B) and (C) Time course of protein expression. (C) Fluorescence in cells infected with the recombinant virus. (TIF) [file pone.0189741.s001.tif]

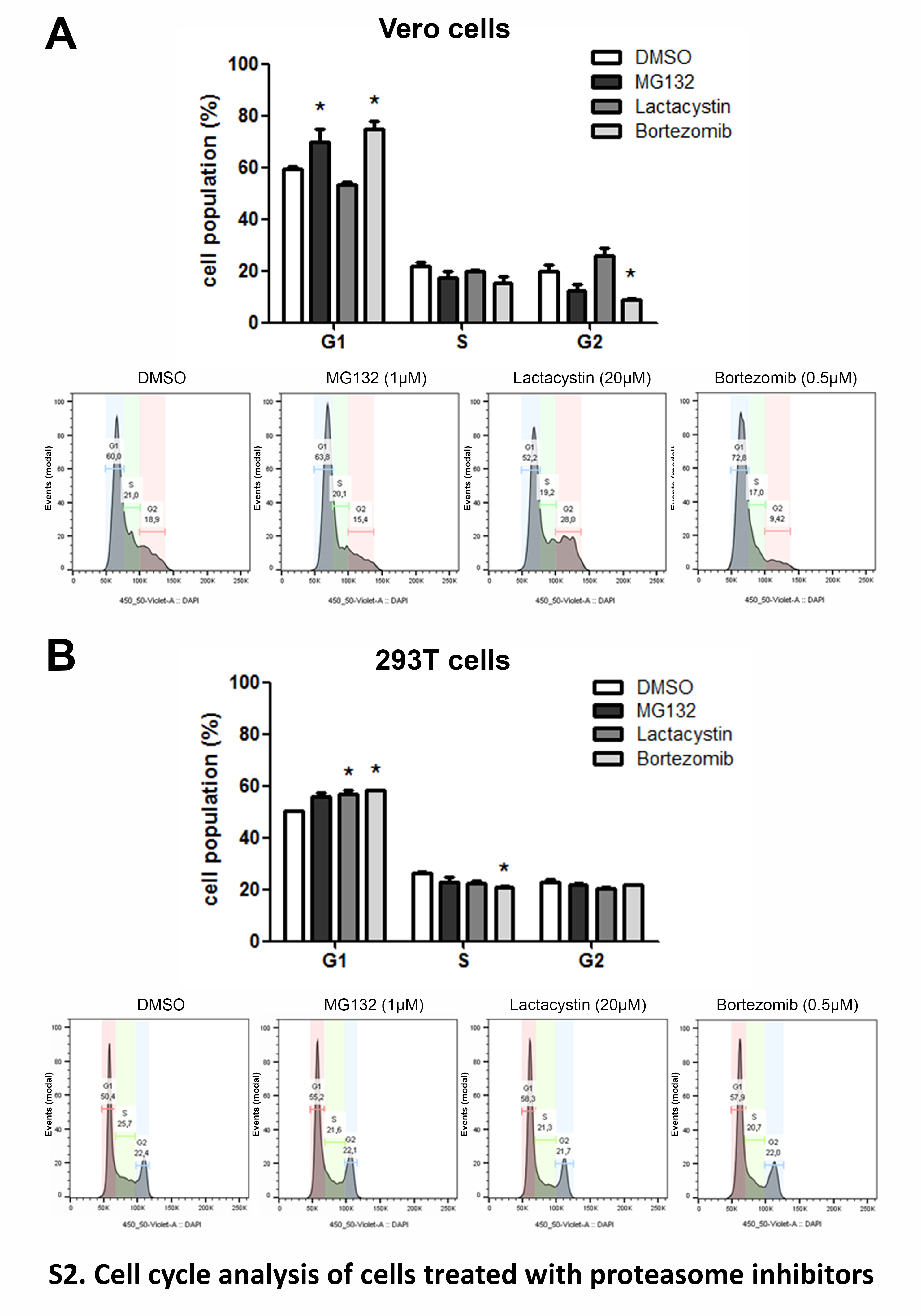

Supplement: S2 Fig — (A) DNA histograms of cell cycle profile and cell cycle phase quantification of Vero cells treated with 1 μM MG132, 20 μM Lactacystin and 0.5 μM Bortezomib for 16h and subjected to cell cycle analysis by flow cytometry with DAPI staining. (B) DNA histograms and cell cycle phase quantification of HEK293T cells treated with 1 μM MG132, 20 μM Lactacystin and 0.5 μM Bortezomib. (TIF) [file pone.0189741.s002.tif]
